# Hydrogen Sulfide (H_2_S)-Donating Formyl Peptide Receptor 2 (FPR2) Agonists: Design, Synthesis, and Biological Evaluation in Primary Mouse Microglia Culture

**DOI:** 10.3390/antiox14070827

**Published:** 2025-07-04

**Authors:** Leonardo Brunetti, Fabio Francavilla, Mauro Niso, Jakub Kosma Frydrych, Ewa Trojan, Igor A. Schepetkin, Liliya N. Kirpotina, Beata Grygier, Krzysztof Łukowicz, Mark T. Quinn, Agnieszka Basta-Kaim, Enza Lacivita, Marcello Leopoldo

**Affiliations:** 1 Dipartimento di Farmacia-Scienze del Farmaco, Università Degli Studi di Bari Aldo Moro, via Orabona 4, 70125 Bari, Italy; leonardo.brunetti@uniba.it (L.B.); fabio.francavilla@uniba.it (F.F.); mauro.niso@uniba.it (M.N.); marcello.leopoldo@uniba.it (M.L.); 2 Laboratory of Immunoendocrinology, Department of Experimental Neuroendocrinology, Maj Institute of Pharmacology, Polish Academy of Sciences, 12 Smętna St., 31-343 Kraków, Poland; frydrych@if-pan.krakow.pl (J.K.F.); trojan@if-pan.krakow.pl (E.T.); grygier@if-pan.krakow.pl (B.G.); lukowicz@if-pan.krakow.pl (K.Ł.); basta@if-pan.krakow.pl (A.B.-K.); 3 Department of Microbiology and Cell Biology, Montana State University, Bozeman, MT 59717, USA; schepetkin@yahoo.com (I.A.S.); kirpotina@hotmail.com (L.N.K.); mquinn@montana.edu (M.T.Q.)

**Keywords:** neuroinflammation, hybrid compounds, antioxidant, neuroprotection, anti-inflammation

## Abstract

Chronic neuroinflammation and oxidative stress play an important role in the onset and progression of neurodegenerative disorders, including Alzheimer’s disease, which can ultimately lead to neuronal damage and loss. The mechanisms of sustained neuroinflammation and the coordinated chain of events that initiate, modulate, and then lead to the resolution of inflammation are increasingly being elucidated, offering alternative approaches for treating pathologies with underlying chronic neuroinflammation. Here, we propose a new multitarget approach to address chronic neuroinflammation and oxidative stress in neurodegenerative disorders by activating the formyl peptide receptor 2 (FPR2) combined with the potentiation of hydrogen sulfide (H_2_S) release. FPR2 is a key player in the resolution of inflammation because it mediates the effects of several endogenous pro-resolving mediators. At the same time, H_2_S is an endogenous gaseous transmitter with anti-inflammatory and pro-resolving properties, and it can protect against oxidative stress. Starting from potent FPR2 agonists identified in our laboratories, we prepared hybrid compounds by embedding an H_2_S-donating moiety within the molecular scaffold of these FPR2 agonists. Following this approach, we identified several compounds that combined potent FPR2 agonism with the ability to release H_2_S. The release of H_2_S was assessed in buffer and intracellularly. Compounds **7b** and **8b** combined potent FPR2 agonist activity, selectivity over FPR1, and the ability to release H_2_S. Compounds **7b** and **8b** were next studied in murine primary microglial cells stimulated with lipopolysaccharide (LPS), a widely accepted in vitro model of neuroinflammation. Both compounds were able to counterbalance LPS-induced cytotoxicity and the release of pro-inflammatory (IL-18, IL-6) and anti-inflammatory (IL-10) cytokines induced by LPS stimulation.

## 1. Introduction

Inflammation is the series of responses that the organism enacts when exposed to infectious or sterile tissue damage. The inflammatory process has the physiological goal of restoring normal tissue function and homeostasis. It is, however, known that dysregulated or unresolved inflammation leads to tissue damage, underlying many chronic inflammatory diseases, and eventually leads to loss of organ function [1]. Unresolved inflammation is a characteristic common to a variety of human conditions, including Alzheimer’s disease (AD) [2], atherosclerosis [3], cardiovascular disease [4], and cancer [5]. It has thus become clear that, to avoid persistent chronic inflammation and ensure an adequate return to homeostasis, it is necessary to promote the resolution of inflammatory processes. Physiologically, this is mediated by pro-resolving mediators. These substances can hinder, or even entirely negate, neutrophil tissue infiltration, but they can also counter-regulate chemokines and cytokines, neutrophil apoptosis (and subsequent efferocytosis by macrophages). Finally, pro-resolving mediators are involved in the reprogramming of macrophages and in the induction of tissue repair [6,7]. Pro-resolving mediators are a very broad category of substances, including, among many others, lipoxin A4 (LXA4) [1] and the gaseous mediator hydrogen sulfide (H_2_S) [8].

LXA4’s biological actions are elicited by activating formyl peptide receptor 2 (FPR2) [9], which is a G protein-coupled receptor expressed by cell types involved in immune processes, such as neutrophils and monocytes/macrophages. This expression pattern is reflected in the central nervous system, where FPR2 is expressed by microglia, but it can also be found in astrocytes and neurons [10,11,12,13].

FPR2 is characterized by complex functional properties. Structurally different agonists can stimulate FPR2 and activate different intracellular signaling pathways, depending on the structure and/or the cell type involved. For example, in monocytes and microglia, the high-affinity FPR2 ligand Aβ_1–42_, once bound to the receptor, is rapidly internalized into the cytoplasm, where transient activation of FPR2 by Aβ stimulates rapid protein degradation. In contrast, chronic FPR2 activation contributes to the formation of fibrillar aggregates. Additionally, Aβ_1–42_-FPR2 interaction is linked to the release of pro-inflammatory mediators [14]. Similarly, FPR2 is responsible for the pro-inflammatory effects elicited by bacterial and mitochondrial *N*-formyl peptides, prion protein PrP106-126, and serum amyloid A [12]. However, as previously mentioned, FPR2 also mediates anti-inflammatory and pro-resolving effects if activated by a specialized pro-resolving mediator such as LXA4 [12,13,14].

In the central nervous system, LXA4 enhances neuronal survival and the phagocytic and anti-inflammatory potential of microglia. For instance, LXA4 regulates M1/M2 polarization through the Notch signaling pathway and changes the balance between pro-inflammatory and anti-inflammatory cytokines in favor of the latter by inhibiting the activation of NF-κB and MAPKs in microglial cells [15,16].

Various studies have shown that FPR2 agonists have anti-inflammatory properties [17]. For instance, the FPR2 agonist MR-39 (compound **3a**, Table 1, [18]), developed by us, exhibited anti-inflammatory properties in both LPS-stimulated rat primary microglial cells and mice organotypic hippocampal cultures, being able to counterbalance LPS effects on pro-inflammatory and anti-inflammatory cytokine levels [19,20]. The observed effects resulted from decreased NLRP3 inflammasome activation and diminished the phosphorylation of the transcription nuclear factor-κB (NF-κB), which, in turn, suppressed the transcription of pro-inflammatory cytokines [20]. Similar results were obtained in mice organotypic hippocampal cultures stimulated with fibrillary Aβ_1–42_ [21]. In vivo administration of **3a** in APP/PS1 mice, a double-transgenic mouse expressing a chimeric mouse/human amyloid precursor protein and a mutant human presenilin 1 (animal model of AD), led to an improvement of neuronal survival and decreased microglial cell density and plaque load [21]. Similarly, chronic administration of MR39 in two mouse models of autism spectrum disorder improved inflammatory markers, with a beneficial effect on social behavior [22]. AMS21 and CMC23 (compounds **3b** and **3c**, Table 1, [23]), two FPR2 agonists structurally related to **3a**, demonstrated beneficial protective and anti-inflammatory properties at nanomolar doses in organotypic hippocampal cultures, thus confirming that FPR2 is a promising target for enhancing the resolution of inflammation [24,25].

In mammals, H_2_S is an endogenous gaseous transmitter, along with nitric oxide (NO) and carbon monoxide. Endogenous H_2_S is enzymatically synthesized from L-cysteine by cystathionine β-synthase (CBS), cystathionine γ-lyase (CSE), and 3-mercaptopyruvate sulfur transferase (3-MST) [8]. These enzymes are present in the peripheral and central nervous systems, with CBS primarily localized in astrocytes [26], CSE primarily in neurons [27,28], and 3-MST in both [29,30,31,32]. A seminal study by Zanardo and coworkers first showed that H_2_S is an important endogenous anti-inflammatory and pro-resolution mediator [33]. Subsequent studies have demonstrated that H_2_S regulates signaling molecules associated with inflammation, including silent information regulator-1 (SIRT1) [34,35], mammalian target of rapamycin (mTOR) [36], and NF-κB [37,38,39]. A practical example of this is the fact that H_2_S can inhibit the inflammatory response elicited by LPS by blocking NF-κB transactivation in endothelial cells [40].

In the brain, H_2_S regulates redox balance. For example, H_2_S modulates oxidative stress by inducing cystine uptake into neurons through the cysteine transporter and the cystine/glutamate antiporter. This, in turn, activates the biosynthesis of glutathione, one of the most abundant endogenous antioxidants in the body [41,42,43]. Multiple pieces of evidence have highlighted the critical role of H_2_S in neuroprotection and normal cognitive function, as well as dysregulated H_2_S homeostasis in neurodegenerative conditions. For example, in AD patients, the expression of CBS is drastically decreased, resulting in reduced plasma H_2_S levels that correlate with the severity of cognitive impairments [44]. It has been proposed that strategies that can lead to measured delivery of H_2_S or are able to boost its production may have therapeutic potential [45]. In particular, developing hybrid molecules that release H_2_S in conjunction with other neuroprotective properties represents an exciting and intriguing task to pursue. Based on such evidence, we designed and characterized a set of hybrid molecules able to activate FPR2 and to simultaneously release H_2_S.

A pharmacologically efficient H_2_S donor should be soluble in aqueous media, not be toxic, and slowly release H_2_S over time. For example, the well-known donor sodium hydrogen sulfide (NaHS) releases H_2_S too quickly, which may cause detrimental effects when administered to living organisms. Over the years, many research efforts have elucidated the biological properties of naturally occurring and synthetic H_2_S donors, emphasizing the exploitation of H_2_S biological signaling in a therapeutic context [46]. Along this line, several H_2_S-donor hybrids have been prepared by linking an H_2_S-releasing moiety, such as 4-hydroxybenzamide (TIA) or the 5-(5-hydroxyphenyl)-3*H*-1,2-dithiol-3-one (ADT-OH), to the structure of a drug through a spacer, proving that increasing H_2_S delivery has beneficial effects in preclinical models of several pathologies characterized by chronic inflammation [47]. The conjugation approach could present limitations because the obtained hybrid molecule often presents poor drug-like properties. In this work, we used a different strategy to obtain H_2_S-donating FPR2 agonists by embedding the H_2_S-releasing moiety within the molecular scaffold interacting with FPR2. To this end, we selected the FPR2 agonists **3a**–**c** (Table 1). We exploited the presence of a urea and amide moiety to obtain the corresponding thiourea (compounds **5a**–**c** and **6b**, Table 1) and thioamide (compounds **7a**,**b**, and **8b**, Table 1) derivatives, which can release H_2_S in aqueous media [48]. It is important to note that, by releasing H_2_S, thioureas **5a**–**c**, **6b**, and thioamides **7a**,**b**, **8b** form the oxygenated counterparts, which are potent FPR2 agonists.

## 2. Materials and Methods

### 2.1. Chemistry

Thin layer chromatography (TLC) was performed using plates from Merck (Darmstadt, Germany) (silica gel 60 F254). Normal phase column chromatography was performed using Merck silica gel 60 Å (63–200 µm; 1:30 (*w*/*w*) crude mixture: silica gel ratio). Flash chromatographic separations were performed using pre-packed silica cartridges (KP-Sil 32–63 μm, 60 Å) on a Biotage SP1 purification system (Biotage AB Sweden, Uppsala, Sweden). H^1^ NMR spectra were recorded on a 500-vnmrs500 Agilent spectrometer (500 MHz) (Agilent, Santa Clara, CA, USA) or a Varian Mercury-VX spectrometer (300 MHz) (Agilent, Santa Clara, CA, USA). Chemical shift values were reported in ppm (δ). Mass spectra were recorded on an HP6890-5973 MSD gas chromatograph/mass spectrometer; significant *m*/*z* peaks, with their percentage of relative intensity in parentheses, are reported. HRMS-ESI spectra were recorded on an Agilent 6530 Accurate Mass MicroQ-TOF (Agilent, Santa Clara, CA, USA). All spectra were in agreement with the assigned structures. Elemental analyses (C,H,N) of the target compounds were performed on a Eurovector Euro EA 3000 analyzer (Eurovector, Pavia, Italy). Analytical HPLC analyses were performed on an Agilent 1260 Infinity Binary LC System equipped with a diode array detector using a Phenomenex Synergi Fusion-RP column (100 mm × 3 mm, 4 μm particle size) (Phenomenex, Torrance, CA, USA). Gradient elution (10 min, phase A: 0.1 % of formic acid in water, phase B: 0.1% formic acid in ACN; the gradient is from 90% to 0% of A) at a flow rate of 0.7 mL/min was used. All compounds exhibited ≥ 95% purity.

The following compounds were prepared according to the literature methods: (*S*)-*t*-butyl [1-[[[1-(3-chloro-4-fluorophenyl)cyclopropyl]methyl]amino]-3-(4-cyanophenyl)-1-oxopropan-2-yl]carbamate (**1a**) [18]; (*S*)-*t*-butyl [3-(4-cyanophenyl)-1-(indolin-1-yl)-1-oxopropan-2-yl]carbamate (**1b**) [23]; (*S*)-*t*-butyl [3-(4-cyanophenyl)-1-(6-fluoroindolin-1-yl)-1-oxopropan-2-yl]carbamate (**1c**) [23]; (*S*)-2-amino-N-[[1-(3-chloro-4-fluorophenyl)cyclopropyl]methyl]-3-(4-cyanophenyl)propenamide (**2a**) [18], (*S*)-4-(2-amino-3-(indolin-1-yl)-3-oxopropyl)benzonitrile (**2b**) [23], (*S*)-4-(2-amino-3-(6-fluoroindolin-1-yl)-3-oxopropyl)benzonitrile (**2c**) [23], (*S*)-N-[[1-(3-chloro-4-fluorophenyl)cyclopropyl]methyl]-3-(4-cyanophenyl)-2-[3-(4-fluorophenyl)ureido]propenamide (**1a**) [18], (*S*)-1-[3-(4-cyanophenyl)-1-(indolin-1-yl)-1-oxopropan-2-yl]-3-(4-fluorophenyl)urea (**1b**) [23], (*S*)-1-[3-(4-cyanophenyl)-1-(6-fluoroindolin-1-yl)-1-oxopropan-2-yl]-3-(4-fluorophenyl)urea (**1c**) [23]; [1-(3-chloro-4-fluorophenyl)cyclopropyl]methanamine (**9a**) [18]; 6-fluoro-indoline (**9c**) [49].

#### 2.1.1. General Procedure for the Synthesis of Compounds **4b**, **5a**–**c**, **6b**

A mixture of the amine (*S*)-**2a**–**c** (0.42 mmol) and the appropriate isocyanate or isothiocyanate derivate (0.46 mmol) in anhydrous THF is stirred at room temperature overnight. At the end, the solvent is evaporated in vacuo and the crude product is purified by flash chromatography using gradient elution from *n*-hexane/EtOAc 8:2 to *n*-hexane/EtOAc 1:1 to obtain the pure desired compound as detailed below.

***(S)*-3-(4-Bromophenyl)-1-[3-(4-cyanophenyl)-1-(indolin-1-yl)-1-oxopropan-2-yl]urea (4b).**

White solid, yield: 45%. ^1^H NMR (500 MHz, DMSO-*d*_6_) δ 2.96–3.00 (m, 1H), 3.06–3.28 (m, 3H), 4.12 (dt, 1H, *J* = 6.8 and 10.3 Hz), 4.29 (dt, 1H, *J* = 7.3 and 10.3 Hz), 4.81 (dd, 1H, *J* = 8.3 and 13.7 Hz), 6.79 (d, 1H, *J* = 8.3 Hz, D_2_O exchanged), 6.99–7.03 (m, 1H), 7.15 (t, 1H, *J* = 7.8 Hz), 7.24 (d, 1H, *J* = 6.9 Hz), 7.29 (d, 2H, *J* = 9.3 Hz), 7.34 (d, 2H, *J* = 9.3 Hz), 7.48 (d, 2H, *J* = 8.3 Hz), 7.74 (d, 2H, *J* = 8.3 Hz), 8.05 (d, 1H, *J* = 7.8 Hz), 8.80 (s, 1H, D_2_O exchanged). HRMS (ESI^−^) calcd for [(C_25_H_21_BrN_4_O_2_)-H]^−^: 487.0770, found 487.0769. ESI^−^/MS/MS [M − H]^−^ *m*/*z* 116 (100), 324 (91), 326 (93).

**(*S*)-N-[[1-(3-Chloro-4-fluorophenyl)cyclopropyl]methyl]-3-(4-cyanophenyl)-2-[3-(4-fluorophenyl)thioureido]propanamide (5a).**

Yellowish solid, yield: 55%. ^1^H NMR (500 MHz, CDCl_3_) δ 0.75–0.84 (m, 4H), 3.09 (dd, 1H, *J* = 8.1 Hz and 14.0 Hz), 3.14 (dd, 1H, *J* = 6.2 Hz and 14.0 Hz), 3.29 (dd, 1H, *J* = 5.8 Hz and 14.0 Hz), 3.39 (dd, 1H, *J* = 5.7 Hz and 14.0 Hz), 5.15 (dd, 1H, *J* = 7.8 Hz and 14.3 Hz), 6.11 (app t, 1H, D_2_O exchanged), 6.35 (d, 1H, *J* = 7.7 Hz, D_2_O exchanged), 6.98–7.02 (m, 2H), 7.04–7.10 (m, 4H), 7.22–7.23 (m, 3H), 7.55 (d, 2H, *J* = 8.2 Hz), 7.68 (br s, 1H, D_2_O exchanged). HRMS (ESI^+^) calcd for [(C_27_H_23_ClF_2_N_4_OS) + Na]^+^: 547.1147, found 547.1141. ESI^+^/MS/MS [M + Na]^+^ *m*/*z* 76 (73), 394 (100).

**(*S*)-1-[3-(4-Cyanophenyl)-1-(indolin-1-yl)-1-oxopropan-2-yl]-3-(4-fluorophenyl)thiourea (5b).**

Yellowish solid, yield: 52%. ^1^H NMR (500 MHz, CDCl_3_) δ 2.97–3.03 (m, 1H), 3.15–3.23 (m, 2H), 3.29 (dd, 1H, *J* = 7.7 Hz and 3.5 Hz), 3.59 (dt, 1H, *J* = 6.8 Hz and 10.2 Hz), 4.36 (dt, 1H, *J* = 6.0 Hz and 10.2 Hz), 5.66 (dd, 1H, *J* = 7.7 Hz and 13.7 Hz), 7.03–7.10 (m, 3H), 7.15–7.21 (m, 4H), 7.34 (d, 2H, *J* = 8.1 Hz), 7.54 (d, 2H, *J* = 8.1 Hz), 7.88 (br s, 1H, D_2_O exchanged) 8.03 (d, 1H, *J* = 8.2 Hz). HRMS (ESI^+^) calcd for [(C_25_H_21_FN_4_OS) + Na]^+^: 467.1318, found 467.1333. ESI^+^/MS/MS [M + Na]^+^ *m*/*z* 76 (100).

**(*S*)-1-(3-(4-Cyanophenyl)-1-[(6-fluoroindolin-1-yl)-1-oxopropan-2-yl]-3-(4-fluorophenyl)thiourea (5c).**

Yellowish solid, yield: 55%. ^1^H NMR (500 MHz, CDCl_3_) δ 2.89–2.95 (m, 1H), 3.04–3.15 (m, 1H), 3.22 (dd, 1H, *J* = 5.6 Hz and 13.4 Hz), 3.27 (dd, 1H, *J* = 8.1 Hz and 13.4 Hz), 3.52–3.57 (m, 1H), 4.38 (dt, 1H, *J* = 5.9 Hz and 10.3 Hz), 5.63 (dd, 1H, *J* = 7.8 Hz and 13.7 Hz), 6.75–6.79 (m, 2H), 7.08–7.17 (m, 2H), 7.33 (d, 2H, *J* = 8.1 Hz), 7.56 (d, 2H, *J* = 8.1 Hz), 7.68 (br s, 1H, D_2_O exchanged), 7.83–7.85 (m, 1H). HRMS (ESI^+^) calcd for [(C_25_H_20_F_2_N_4_OS) + Na]^+^: 485.1224, found 485.1221. ESI^+^/MS/MS [M + Na]^+^ *m*/*z* 76 (100).

**(*S*)-1-(4-Bromophenyl)-3-[3-(4-cyanophenyl)-1-(indolin-1-yl)-1-oxopropan-2-yl]thiourea (6b).**

Yellowish solid, yield: 79%. ^1^H NMR (300 MHz, CDCl_3_) δ 2.97–3.07 (m, 1H), 3.15–3.32 (m, 3H), 3.62 (dt, 1H, *J* = 6.7 Hz and 10.3 Hz), 4.39 (dt, 1H, *J* = 6.2 Hz and 10.3 Hz), 5.64 (dd, 1H, *J* = 7.3 Hz and 13.8 Hz), 7.04–7.12 (m, 3H), 7.15–7.23 (m, 2H), 7.35 (d, 2H, *J* = 8.3 Hz), 7.43 (d, 2H, *J* = 8.7 Hz), 7.55 (d, 2H, *J* = 8.3 Hz), 7.99 (br s, 1H, D_2_O exchanged), 8.02–8.05 (m, 1H). HRMS (ESI^+^) calcd for [(C_25_H_21_BrN_4_OS) + Na]^+^: 527.0517, found 527.0517. ESI^+^/MS/MS [M + Na]^+^ *m*/*z* 247 (100).

#### 2.1.2. General Procedure for the Synthesis of the Target Compounds **7a**,**b** and **8b**

Lawesson’s reagent (0.049 mmol) is added to a solution of the amide (*S*)-**3a**,**b**, and **4b** (0.06 mmol) in anhydrous toluene (5 mL). The reaction mixture is heated at 100 °C for 2–3 h. Then, the solvent is evaporated under reduced pressure, and the crude product is purified by chromatography to obtain the pure desired compound as detailed below.

**(*S*)-*N*-[[1-(3-Chloro-4-fluorophenyl)cyclopropyl]methyl]-3-(4-cyanophenyl)-2-[3-(4-fluorophenyl)ureido]propanethioamide (7a).**

Eluted with *n*-hexane/EtOAc 7:3. Yellowish solid, yield: 95%. ^1^H NMR (500 MHz, CDCl_3_) δ 0.77–0.83 (m, 3H), 0.86–0.89 (m, 1H), 3.06 (dd, 1H, *J* = 7.5 Hz and *J* = 13.6 Hz), 3.12 (dd, 1H, *J* = 7.1 Hz and *J* = 13.6 Hz), 3.59 (dd, 1H, *J* = 4.6 Hz and *J* = 14.5 Hz), 3.79 (dd, 1H, *J* = 5.8 Hz and *J* = 14.5 Hz), 4.84 (dd, 1H, *J* = 7.5 Hz and *J* = 15.7 Hz), 5.81 (d, 1H, *J* = 8.5 Hz, D_2_O exchanged), 6.71 (br s, 1H, D_2_O exchanged), 6.95–7.02 (m, 4H), 7.11–7.13 (m, 2H), 7.19 (dd, 1H, *J* = 2.2 Hz adn *J* = 6.9 Hz), 7.24 (d, 2H, *J* = 8.3 Hz), 7.51 (d, 2H, *J* = 8.3 Hz), 8.42 (br s, 1H, D_2_O exchanged). HRMS (ESI^+^) calcd for [(C_27_H_23_ClF_2_N_4_OS) + Na]^+^: 547.1147, found 547.1143. ESI^+^/MS/MS [M + Na]^+^ *m*/*z* 76 (100),185 (90).

**(*S*)-1-[3-(4-Cyanophenyl)-1-(indolin-1-yl)-1-thioxopropan-2-yl]-3-(4-fluorophenyl)urea (7b).**

Eluted with *n*-hexane/EtOAc 1:1. Yellowish solid, yield: 48%. ^1^H NMR (300 MHz, CDCl_3_, mixture of isomers 1:0.8) δ 2.54–2.67 (m, 0.53H), 2.76–2.92 (m, 1.08H), 3.03–3.21 (m, 2.11H), 3.30 (dd, 0.54H, *J* = 8.0 Hz and *J* = 13.2 Hz), 3.65–3.75 (m, 0.61H), 4.09–4.21 (m, 0.68H), 4.48–4.56 (m, 0.46H), 4.59–4.68 (m, 0.50H), 5.38–5.46 (m, 0.52H), 6.16 (d, 0.43H, *J* = 5.6 Hz, D_2_O exchanged), 6.19 (d, 0.51H, *J* = 5.6 Hz, D_2_O exchanged), 6.25–6.33 (m, 0.45H), 6.75 (br s, 0.52H, D_2_O exchanged), 6.78 (br s, 0.38H, D_2_O exchanged), 6.95–7.04 (m, 2H), 7.08–7.25 (m, 4.59H), 7.27–7.32 (m, 1.03H), 7.32–7.35 (m, 0.85H), 7.37–7.39 (m, 1.11H), 7.76 (d, 0.45H, *J* = 8.2 Hz), 9.32–9.35 (m, 0.54H). HRMS (ESI^+^) calcd for [(C_25_H_21_FN_4_OS) + Na]^+^: 467.1318, found 467.1315. ESI^+^/MS/MS [M + Na]^+^ *m*/*z* 76 (100).

**(*S*)-1-(4-Bromophenyl)-3-[3-(4-cyanophenyl)-1-(indolin-1-yl)-1-thioxopropan-2-yl]urea (8a).**

Eluted with *n*-hexane/EtOAc 1:1. Yellowish oil, yield: 92%. ^1^H NMR (500 MHz, CDCl_3_, mixture of isomers 1:0.8) δ 2.58–2.65 (m, 0.49H), 2.79–2.91 (m, 1.02H), 3.08–3.14 (m, 1H), 3.15–3.21 (m, 1.02H), 3.29–3.33 (m, 0.71H), 3.67–3.75 (m, 0.58H), 4.15–4.20 (m, 0.50H), 4.50–4.55 (m, 0.45H), 4.58–4.64 (m, 0.51H), 5.38–5.43 (m, 0.51H), 6.12–6.18 (m, 0.90H, D_2_O exchanged), 6.26–6.31 (m, 0.45H), 6.63 (br s, 0.45H, D_2_O exchanged), 6.66 (br s, 0.43H, D_2_O exchanged), 7.10–7.13 (m, 0.45H), 7.16–7.21 (m, 2.96H), 7.23–7.25 (m, 2.36H), 7.34–7.36 (m, 0.94H), 7.37–7.42 (m, 3H), 7.52–7.54 (m, 1.05H), 7.74 (d, 0.52H, *J* = 8.3 Hz), 9.32–9.34 (m, 0.52H).HRMS (ESI^+^) calcd for [(C_25_H_21_BrN_4_OS) + Na]^+^: 527.0517, found 527.0511. ESI^+^/MS/MS [M + Na]^+^ *m*/*z* 128 (100), 246 (78).

### 2.2. Cell-Free H_2_S Release Studies

#### 2.2.1. Fluorescence Assay

The H_2_S release fluorescence assay was carried out in a final volume of 100 μL PBS/DMSO 10% in black 96-well plates. Solutions with decreasing concentrations of each tested compound were prepared in PBS/DMSO 10%. Then, 50 μL of each solution was dispensed in triplicate in each well. Starting from a 10 mM stock solution, a 200 μM solution of probe **9** was prepared and stored in the dark until 50 μL was dispensed in each well. Blank wells containing only the probe at 100 μM in 100 μL of PBS/DMSO 10% are also prepared. A H_2_S calibration curve was obtained by incubating solutions of NaSH with the probe under the same conditions, although a different plate was used to minimize interferences due to the diffusion of rapidly released H_2_S. After 24 h and 48 h incubation in a humidified incubator at 37 °C, 5% CO_2_, fluorescence reads are obtained from each well with a plate reader TECAN M1000 Pro (Tecan, Männedorf, Switzerland), at λ_ex_ = 350 nm and λ_em_ = 440 nm. Each experiment was replicated at least three times. The data were processed using Microsoft Excel (Microsoft, Redmond, WA, USA) and GraphPad Prism 7 (GraphPad Software, La Jolla, CA, USA).

#### 2.2.2. MTT-Based Assay

Solutions at different concentrations of each compound in 100 µL of serum-free MEM/10% DMSO with 0.5 mg/mL 3-(4,5-dimethylthiazol-2yl)-2,5-diphenyltetrazolium bromide (MTT) were incubated for 5 h or 24 h in a humidified incubator at 37 °C in 5% CO_2_ atmosphere. The experimental design comprised blank wells containing only medium and MTT, and a H_2_S calibration curve obtained by incubating nine solutions at decreasing concentrations (100 µM to 0.01 µM) of NaHS in medium and MTT. At the end of incubation time, the supernatant was removed, the tetrazolium crystals were dissolved in 100 µL DMSO/EtOH (1:1 *v*/*v*), and absorbance values were read using a Victor 3 plate reader (PerkinElmer, Shelton, CT, USA) at 570 nm. H_2_S values are expressed as mean ± S.E.M. of the results of three independent experiments conducted in triplicate, and were plotted using GraphPad Prism 7.

### 2.3. Biological Methods

#### 2.3.1. Ca^2+^ Mobilization Assay

As previously reported, intracellular calcium concentrations [Ca^2+^] were measured using a FlexStation II scanning fluorometer (Molecular Devices, Sunnyvale, CA, USA). The cells were suspended in Hank’s balanced salt solution without Ca^2+^ and Mg^2+^ but with 10 mM HEPES (HBSS^−^), loaded with 1.25 μg/mL Fluo-4 AM dye, and incubated at 37 °C for 30 min in the dark. After loading of the dye, the cells were washed with HBSS^−^ containing 10 mM HEPES, resuspended in HBSS^+^ containing Ca^2+^, Mg^2+^, and 10 mM HEPES (HBSS^+^), and aliquoted in flat bottom, half-area-well black microtiter plates (2 × 10^5^ cells/well). The evaluation of direct agonist activity was performed by adding test compounds from a source plate containing test dilutions in HBSS^+^, and changes in fluorescence were monitored (λ_ex_ = 485 nm, λ_em_ = 538 nm) every 5 s for 240 s at room temperature after compound addition. Maximum change in fluorescence during the first 3 min, expressed in arbitrary units over baseline, was used to determine a response. Responses for FPR2 agonists were normalized to the response of 5 nM WKYMVM in FPR2-HL60 cells, which was assigned a value of 100%. Responses for FPR1 agonists were normalized to the response induced by 5 nM *f*MLF in FPR1-HL60 cells. To evaluate the inhibitory effects of the compounds on FPR2- or FPR1-dependent Ca^2+^ flux, test compounds were added to the wells (final DMSO concentration was 1%) containing FPR2- or FPR1-HL60 cells. The samples were preincubated for 10 min, then 5 nM WKYMVM (for FPR2-HL60 cells) or 5 nM *f*MLF (for FPR1-HL60 cells) was added. The maximum change in fluorescence, expressed in arbitrary units over the baseline, was used to determine the agonist response. Curve fitting (at least five or six points) and calculation of median effective concentration values (EC_50_ or IC_50_) were performed by nonlinear regression analysis of the dose–response curves generated using Prism 9 (GraphPad Software, Inc., San Diego, CA, USA). Efficacy was assessed by comparing the response induced by the test compounds to that induced by a positive control (5 nM WKYMVM for FPR2 or 5 nM fMLF for FPR1), which was assigned a value of 100%.

#### 2.3.2. Cell Viability Assays

The cytotoxicity of our compounds was assessed using the well-established MTT assay. HepG2 hepatocarcinoma cells were cultured in Minimum Essential Medium (MEM) (Euroclone, Pero, Italy), supplemented with 10% fetal bovine serum (Euroclone, Pero, Italy), 100 U/mL penicillin, and 100 µg/mL streptomycin sulfate, at 37 °C in a humidified incubator in 5% CO_2_ atmosphere. For the MTT assays, these cells were cultured up to about 90% confluence and were then seeded at a concentration of 20,000 cells per well in 96-well plates. After 24 h, the culture medium was replaced with solutions at various concentrations of each compound in MEM/DMSO 5% (*v*/*v*). Control wells were incorporated in the experimental design, consisting of vehicle (MEM/DMSO 5%) and pure medium controls.

In the first round of cytotoxicity assays, 0.5 mg/mL MTT was added to each well 2 or 24 h from treatment and left to incubate for 3–5 h. In the second round of cytotoxicity assays, at 2 or 24 h after treatment, treated media were removed, the cells were washed once in PBS, and fresh medium containing 0.5 mg/mL MTT was added to each well and left to incubate for 3–5 h.

Subsequently, the supernatant was removed, and the formazan crystals were dissolved in DMSO/EtOH 1:1 (100 µL/well). Absorbance values were then recorded from each well using a Victor 3 plate reader (PerkinElmer, Shelton, CT, USA) at 570 nm. All presented data were obtained from 3 to 5 experiments performed in triplicate. Cell viability values are expressed as mean ± S.E.M. and were plotted using GraphPad Prism 7.

#### 2.3.3. Intracellular H_2_S Release Studies

All cell culture reagents were purchased from S.I.A.L. S.r.l. (Rome, Italy). WSP-1 (Washington State Probe-1, 3′-methoxy-3-oxo-3H-spiro[isobenzofuran-1,9′-xanthen]-6′-yl 2-(pyridine-2-yl-disulfanyl benzoate), was obtained from Cayman Chemical (Cayman, Ann Arbor, MI, USA). HepG2, human hepatocellular carcinoma cells, was obtained from American Type Culture Collection (ATCC, Bethesda, MD, USA). Human hepatocellular carcinoma cells (HepG2) were cultured in Minimum Essential Eagle’s Medium. This medium was supplemented with 10% (*v*/*v*) Fetal Bovine Serum, 1% (*v*/*v*) Glutamine and 1% (*v*/*v*) Penicillin-Streptomycin, 1% non-essential amino acids. Cells were cultivated at 37 °C with 5% CO_2_ at saturated humidity.

These experiments were carried out as already described [50]. HepG2 cells were cultured up to about 90% confluence, and 24 h before the experiment cells were seeded into a 96-well flat bottom black plate (50,000 cells per well). After 24 h, the medium was replaced and cells were incubated at 37 °C for 30 min with a 10 mM solution of the fluorescent WSP-1 probe, which is highly sensitive for H_2_S detection [51]. After incubation, the supernatant was removed and replaced with different concentrations (100, 200, and 300 µM) of the tested compounds dissolved in HBSS buffer. The change in fluorescence, read with a TECAN M1000 Pro spectrofluorometer (Tecan, Mannedorf, Switzerland, excitation and emission wavelengths of 465 nm and 515 nm, respectively), was monitored every 5 min for 60 min. ADT 100 µM was used as a slow H_2_S-donor reference compound. Data were analyzed by one-way ANOVA for repeated measures, followed by post hoc Bonferroni’s multiple comparison test. Results are expressed as mean ± SEM of 3 independent experiments in triplicate. Statistical significance was accepted at a level of *p* < 0.05.

### 2.4. Mouse Primary Microglial Cell Cultures

#### 2.4.1. Animals

Mice (C57BL/6J) were maintained under standard laboratory conditions (ambient temperature: 23 °C; 12:12 h light/dark cycle with lights on at 08:00; food and water ad libitum). The estrous cycle of female mice was monitored via daily collection and cytological analysis of vaginal smears. Females identified in the proestrus phase were paired with males for a 12 h mating window. The presence of spermatozoa in post-coital vaginal smears was used as confirmation of successful copulation. Pregnant females were subsequently left undisturbed in their home cages for the remainder of gestation. All experimental procedures were approved by the Local Ethics Committee in Kraków, Poland (approval no. 223/2023, dated 9 November 2023).

#### 2.4.2. Cell Culture

Primary microglial cell cultures were derived from the cortices of 1–2-day-old mouse pups (1pup/1 brain/1 flask). After decapitation, the brains were extracted and immediately placed in cold Hanks’ Balanced Salt Solution (HBSS, Gibco, New York, NY, USA) on ice. The brains were washed at least three times with 7 mL of HBSS and then transferred to 10 mL of fresh culture media consisting of Dulbecco’s Modified Eagle Medium (DMEM) with GlutaMax and high glucose (4.5 g/L) (Gibco, New York, NY, USA) on ice. The tissue was minced and incubated in HBSS containing glucose, bovine serum albumin (BSA; Sigma-Aldrich, St. Louis, MO, USA), and HEPES (Gibco, New York, NY, USA) with 0.025% trypsin at 37 °C for 20 min. The trypsinization was halted by adding DMEM with GlutaMax and high glucose (4.5 g/L) supplemented with 10% heat-inactivated fetal bovine serum (FBS, Gibco, New York, NY, USA). The tissue was then gently triturated to obtain a single-cell suspension. Cells were plated at a density of 3 × 10^5^ cells/cm^2^ in culture medium composed of DMEM with GlutaMax and high glucose (4.5 g/L), supplemented with 10% heat-inactivated FBS, 100 U/mL penicillin, and 0.1 mg/mL streptomycin (all from Gibco, New York, NY, USA), in poly-L-lysine-coated 75 cm^2^ culture flasks. On the ninth day in vitro (maintained at 37 °C, 5% CO_2_), the flasks were agitated on a horizontal shaker for 1 h at 37 °C and 80 rpm. Following centrifugation, the cells were resuspended in culture medium and seeded at final densities of 1.25 × 10^6^ cells/well in 6-well plates, 2 × 10^5^ cells/well in 24-well plates, or 4 × 10^4^ cells/well in 96-well plates. The purity of microglial cell cultures was assessed as we have shown previously [20].

#### 2.4.3. Cell Treatment

Microglia cell cultures were pretreated with the FPR2 agonists **8b** (1 µM) or **7b** (1 µM) for 1 h and then stimulated with lipopolysaccharide (LPS; 0.1 μg/mL; Escherichia coli 0111:B4; Sigma-Aldrich, St. Louis, MO, USA) for 24 h. The concentration of 1 μM was chosen based on our preliminary data (Supplementary File). Control cultures were treated with the appropriate vehicle. Stock solutions of the test compounds were prepared as follows: **8b** and **7b** (1 mM DMSO), and LPS (1 mg/mL PBS). The final solutions of the tested compounds were prepared in distilled water. Each experimental set of the control cultures was supplemented with the appropriate vehicles, and the solvent was present in cultures at a final concentration of 0.1% (*v*/*v*).

#### 2.4.4. NO Release Assay

NO (nitrite ion in solution) production from LPS-treated microglial cells was measured using the Griess reaction, as previously described by [20]. After 24 h of treatment, equal volumes of cell culture medium and Griess reagents (Griess A: 0.1% *N*-1-naphthylethylenediamine dihydrochloride and Griess B: 1% sulfanilamide in 5% phosphoric acid) were combined in a 96-well plate and the absorbance was read at 540 nm using an Infinite M200PRO microplate reader (TECAN, Männedorf, Switzerland). The results were normalized to the NO levels released by vehicle-treated cells (set at 100%) and expressed as a percentage of the control ± SD.

#### 2.4.5. Lactate Dehydrogenase (LDH) Release Assay

LDH release was measured as previously described by [18] as an estimation of cell damage 24 h after LPS treatment. Cell culture supernatants were incubated with the reagent mixture according to the manufacturer’s instructions (Cytotoxicity Detection Kit, Roche, Mannheim, Germany). Absorbance was measured at 540 nm using an Infinite M200PRO microplate reader (TECAN, Männedorf, Switzerland). The results were normalized to the LDH activity released from vehicle-treated cells (considered as 100%) and expressed as a percentage of the control ± SD.

#### 2.4.6. Enzyme-Linked Immunosorbent Assay (ELISA)

To assess the effect of test compounds on IL-18, IL-6, and IL-10 levels, the medium of microglial cell cultures was collected 24 h after LPS treatment. The protein levels of the cytokines IL-18 (Mouse interleukin 18 ELISA kit), IL-6 (Mouse interleukin 6 ELISA kit), and IL-10 (Mouse interleukin 10 ELISA kit; all obtained from FineTest, Wuhan, China) were measured using commercially available enzyme-linked immunosorbent assay kits according to the manufacturer’s instructions. The detection limits were as follows: IL-18, 7.5 pg/mL; IL-6, 9.35 pg/mL; and IL-10, 9.35 pg/mL. The precision of both intra- and inter-assay measurements varied depending on the specific attributes of the assay.

#### 2.4.7. Statistical Analysis

The results from primary microglial cell cultures were derived from 2 independent primary microglial cultures, while “n” for each culture was “2–5”. The results for NO/LDH release are expressed as the mean ± SEM (vehicle-treated cells). Data from the ELISA study are reported as the mean ± SEM. Comparisons between groups were made using factorial analysis of variance (ANOVA), followed by Duncan’s post hoc test to identify differences between treatment groups. Although this method is known to be less conservative, it was selected due to its sensitivity in detecting differences in settings with low variability and small group sizes. A *p*-value of 0.05 or less was considered statistically significant. Symbols indicate the following: * *p* < 0.05 vs. control, # *p* < 0.05 vs. LPS group.

## 3. Results and Discussion

### 3.1. Chemistry

**Scheme 1:**
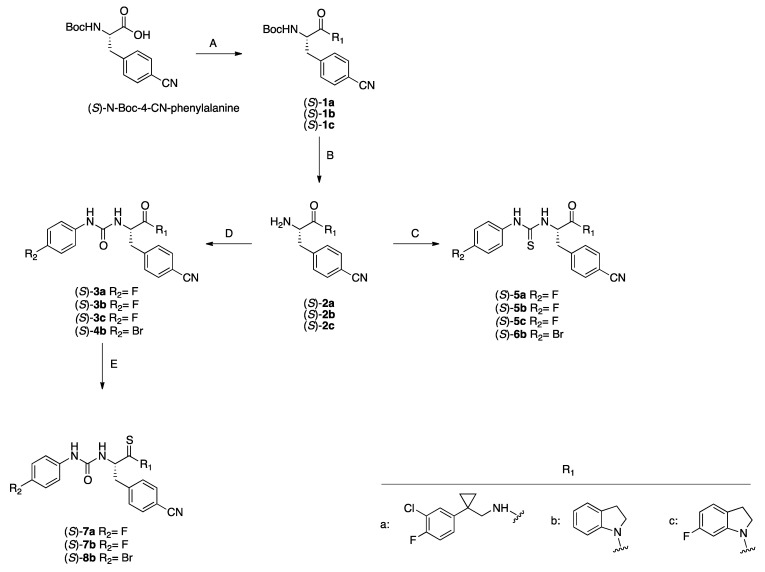
Reagents and conditions: (A) amine **9a**–**c**, PyBOP, *N*-methylmorpholine, r.t.; overnight; 45–87% yield; (B) CF_3_COOH, r.t.; 5 h, quantitative yield; (C) 4-F- or 4-Br-phenylthioisocyanate, r.t.; overnight; 79–55% yield; (D) 4-F- or 4-Br-phenylisocyanate, r.t.; overnight; 79–40% yield; (E) Lawesson’s reagent; 100 °C, 3 h; 50–80% yield.

Synthesis of the target compounds is depicted in Scheme 1. (*S*)-*N*-Boc-4-cyano-phenylalanine was condensed with the appropriate amine using PyBOP as a condensing agent in the presence of *N*-methylmorpholine to obtain the N-Boc derivatives (*S*)-**1a**–**c**. Subsequent deprotection with trifluoroacetic acid gave the key amines (*S*)-**2a**–**c** [18,23]. Thiourea derivatives (*S*)-**5a**–**c** and (*S*)-**6b** were obtained by condensing amines (*S*)-**2a**–**c** with the appropriate 4-substituted phenylthioisocyanate. Thioamide derivatives (*S*)-**7a**–**b** and (*S*)-**8b** were synthesized by condensing amines (*S*)-**2a**–**b** with the appropriate 4-substituted phenylisocyanate to obtain the intermediates amides (*S*)-**3a**–**b** and (*S*)-**4b**, which were converted to the target compounds after thionation with Lawesson’s reagent.

### 3.2. Agonist Activity at FPR2

We assessed the ability of the target compounds to activate FPR2 by measuring their ability to induce Ca^2+^ mobilization in HL-60 cells transfected with human FPR2. Table 1 reports the compounds’ potencies expressed as EC_50_ values. As discussed, our reference FPR2 agonists were **3a**–**c** (Table 1). In addition, we also synthesized a close structural analogue of **3b** in which a 4-bromo substituent replaced a 4-fluoro (derivative **4b**). Even though literature data suggested that such replacement could benefit FPR2 agonist potency [52], we found that **4b** was less potent than **3b**. As for urea/thiourea switch in **3a**–**c**, and **4b**, the activity data showed that it was detrimental for FPR2 agonist potency. In fact, **5a** (the thiourea analog of **3a**) was inactive, while **5b**,**c**, and **6b** were more than 10-fold less potent than **3b**,**c**, and **4b**, respectively. These data further corroborated the urea moiety’s important role in interacting with the FPR2 binding site [53]. The next step was the evaluation of the amide/thioamide switch in **3a**,**b**, and **4b**. This modification resulted in a substantial increase in FPR2 agonist potency in the case of thioamides **7a** and **8b** compared to the corresponding amides **3a** and **4b**, respectively. In contrast, the thioamide **7b** was 23-fold less potent at FPR2 than the corresponding amide **3b**. We could not obtain the thioamide analogue of **3c** in a purity degree suitable for biological assay.

The newly synthesized compounds were counterscreened at FPR1 by measuring their ability to induce Ca^2+^ mobilization in HL-60 cells transfected with human FPR1 (Table 1). Of note, the thiourea or thioamide derivatives were completely inactive at FPR1, resulting in an exceptional improvement in selectivity for FPR2 compared to the corresponding urea and amide derivatives. In particular, the thioamides **7b** and **8b** stood out as potent and selective FPR2 agonists. Finally, we assessed the ability of compounds to induce receptor desensitization at FPR2 by measuring the inhibition of Ca^2+^ mobilization induced by subsequent treatment with a standard FPR2 agonist (IC_50_, Table 1) because FPR2 undergoes homologous desensitization after stimulation with agonists, resulting in functional antagonism (IC_50_, Table 1) [54]. The thioamide derivatives **7a**,**b**, and **8b** induced FPR2 desensitization at submicromolar concentration, while the thioureas were less potent (**6b**, IC_50_ = 2.2 μM) or inactive (**5b** and **5c**). We also evaluated the effects of the thiourea and thioamide derivatives on Ca^2+^ mobilization in FPR1-HL60-transfected cells after stimulation with the FPR1 agonist (IC_50_, Table 1). The compounds were found to be inactive, thus confirming that they did not interact with FPR1.

**Table 1:** Effect of the compounds on Ca^2+^ mobilization in FPR1- and FPR2-HL60-transfected cells (EC_50_) and on FPR1 and FPR2 desensitization (IC_50_).

				Ca^2+^ Mobilization			
				HL60-FPR2		HL60-FPR1	
				EC_50_, μM (Efficacy, %)	IC_50_, μM	EC_50_, μM (Efficacy, %)	IC_50_, μM
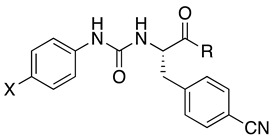							
**Compd**	**X**	**R**					
**3a**	F	a		3.9 *^a^*	N.T. *^b^*	5.2 *^a^*	N.T.
**3b**	F	b		0.026 *^c^*	0.01 *^c^*	0.32 *^c^*	17.6 *^c^*
**3c**	F	c		0.16 *^c^*	0.21 *^c^*	4.8 *^c^*	N.A. *^d^*
**4b**	Br	b		0.10 ± 0.04 (120)	0.35	4.8 ± 1.2 (120)	N.A. *^c^*
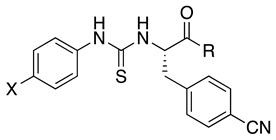							
**5a**	F	a		N.A.	N.A.	N.A.	N.A.
**5b**	F	b		2.2 ± 0.7 (65)	N.A.	N.A.	N.A.
**5c**	F	c		12.0 ± 32 (105)	N.A.	N.A.	N.A.
**6b**	Br	b		1.7 ± 0.7 (65)	2.2 ± 0.4	N.A.	N.A.
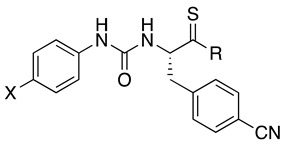							
**7a**	F		a	2.3 ± 0.8 (145)	0.3 ± 0.10	N.A.	N.A.
**7b**	F		b	0.6 ± 0.2 (170)	0.06 ± 0.02	N.A.	N.A.
**8b**	Br		b	0.04 ± 0.02 (150)	0.007 ± 0.003	N.A.	N.A.
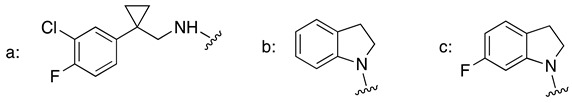							

*^a^* Data taken from [18]; *^b^* N.T.: not tested; *^c^* data taken from [23]; *^d^* N.A.: not active.

### 3.3. Quantification of H_2_S Release by Target Compounds

**Scheme 2:**
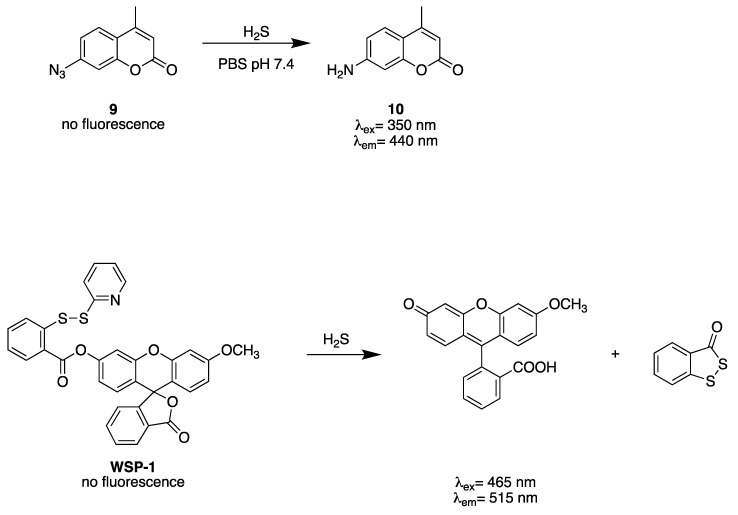
Activation mechanisms by H_2_S of fluorescent probe **9** and WSP-1.

Several methodologies have been reported for H_2_S measurement, which allow for the matching of sample types, specific environments (aqueous media, biological samples, in vivo applications), and experimental requirements with the quantification of H_2_S, such as fluorescence-based and amperometric methods. In this study, we used activity-based fluorescent probes, which exploit an H_2_S-specific chemical reaction to generate a fluorescent response from a specifically designed fluorogenic system, thus allowing H_2_S detection in complex environments such as live cells or tissue cultures with high spatiotemporal resolution [55]. We selected 7-azido-4-methylcoumarin (compound **9**, Scheme 2) for quantifying H_2_S release in a cell-free aqueous medium and the WSP-1 probe (Scheme 2) to measure intracellular H_2_S release.

**Figure 1:**
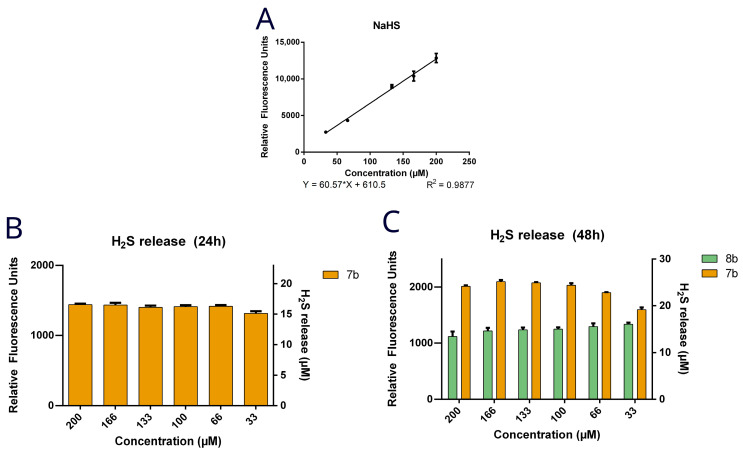
Calibration curve of fluorescent signal emitted by compound **9** using NaHS as source of H_2_S (**A**). H_2_S release detected with probe **9** in aqueous media after 24 h (**B**) and 48 h (**C**). Only compounds **7b** and **8b**, which showed signals above the limit of quantification, were included in the graphs.

Compound **9** is a turn-on fluorescent probe that features an azido moiety which can be reduced to amine by H_2_S, leading to the formation of the fluorescent derivative 7-amino-4-methylcoumarin **10** (λ_ex_ = 350 nm and λ_em_ = 440 nm, Scheme 2). We included NaHS in the assay as a positive control and **3a** as a negative control. Probe **9** (100 μM) was capable of responding to H_2_S released by NaHS in a linear fashion in the range from 30 μM to 200 μM (Figure 1), while it showed no response to compound **3a**. After 24 h incubation with compounds **5a**–**c**, **6b**, **7a**–**c**, **8b**, probe **9** detected H_2_S release only by the thioamide derivative **7b**. After 48 h, the fluorescent signal linked to compound **7b** became more intense, and compound **8b** also started showing a limited capability to release H_2_S (Figure 1). These results indicated that our sulfurated derivatives were relatively stable in aqueous media and do not release H_2_S (except **7b** and, to a lesser degree, **8b**).

Next, we assessed H_2_S release in a biological environment using human hepatocarcinoma HepG2 cells as a model. The intracellular release of H_2_S was detected with the WSP-1 probe, a turn-on fluorescent probe that selectively and irreversibly reacts with H_2_S. Considering that a high micromolar concentration of the test compounds is required to obtain a robust fluorescent signal, we first evaluated the compounds’ cytotoxicity after 2 h and 24 h of incubation with the MTT assay in HepG2 cells. Given the low solubility of our compounds at high micromolar concentrations, we had to increase DMSO concentrations up to 5% in the cell culture medium and this could have an impact on cell viability. We accounted for this by adding a vehicle control with 5% DMSO and a control without DMSO to our experimental design.

**Figure 2:**
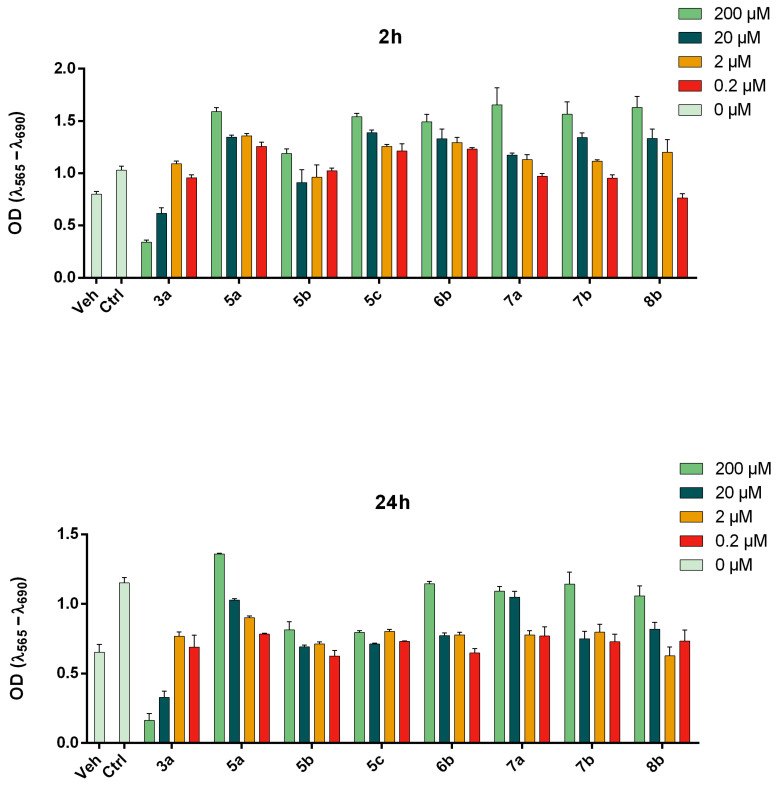
Results of MTT assays performed on HepG2 cells after 2 h and 24 h incubation. Veh, cells with 5% DMSO; Ctrl, cells without DMSO.

As shown in Figure 2, the reference nonsulfurated compound **3a** was cytotoxic (>50% mortality compared to vehicle) at the highest tested doses (200 and 20 µM). In contrast, high doses of the thioureas and thioamides were not cytotoxic and even protected cells from 5% DMSO in the culture media. In fact, we observed absorbance signals higher than the control without DMSO after 2 h incubation with thioamides **7a**–**b** and **8b**. This effect was less pronounced after 24 h incubation. The presence of thioureas in the culture media produced more varied effects compared to the control: while **5a**, **5c**, and **6b** gave a pronounced signal after 2 h incubation, **5a** and **6b** also afforded an absorbance signal higher than the control even after 24 h incubation.

**Figure 3:**
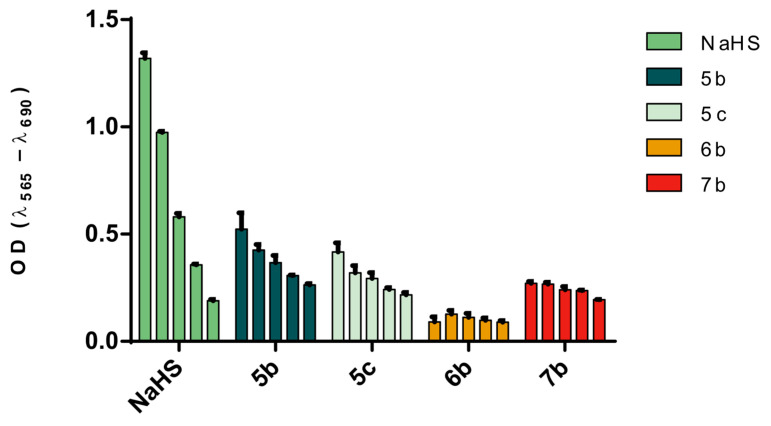
H_2_S release assessed using 3-[4,5-dimethylthiazol-2-yl]-2,5-diphenyl tetrazolium bromide (MTT) as H_2_S-sensing reagent.

To decipher such results and considering the redox properties of H_2_S, we questioned if H_2_S released by our compounds in the culture media could interfere with the conversion of MTT (3-[4,5-dimethylthiazol-2-yl]-2,5-diphenyl tetrazolium bromide) to formazan, which is usually related to cell viability. Thus, we ran cell-free experiments in which MTT was used as the substrate to capture the reductive action of H_2_S. We found, in agreement with Gerő et al. [56], that some of our thioureas and thioamides reduced MTT to formazan after 24 h incubation in MEM with 10% DMSO. The positive control NaHS produced a strong dose-dependent signal, while the negative control **3a** gave no signal, confirming that H_2_S mediated formazan formation (Figure 3).

Unlike the experiments performed with probe **9**, these experiments allowed us to assess whether our sulfurated compounds can release H_2_S in aqueous media. In fact, thioamide **7b** exhibited a robust absorbance signal, indicative of H_2_S release, although this effect was not dose-dependent. In contrast, **7a** and **8b** gave an absorbance signal below the assay’s detection limit. Instead, thioureas **5b** and **5c** showed a significant absorbance signal with slight dose-dependent effects. Compound **6b** behaved like **5b** and **5c** but to a much lesser extent, while **5a** did not give off any detectable absorbance signal. We noted that the (1-(3-chloro-4-fluorophenyl)cyclopropyl)methanamino moiety common to **5a** and **7a** seemed to stifle H_2_S release, while the isoindoline substituent proved more conducive to this activity. In particular, it appears that, with the substituent on the amide being equal, a fluorine atom in the para position to the phenylureido or phenylthioureido moiety is also somewhat advantageous for H_2_S release, as demonstrated by comparing **6b** and **8b** to **5b** and **7b**.

**Figure 4:**
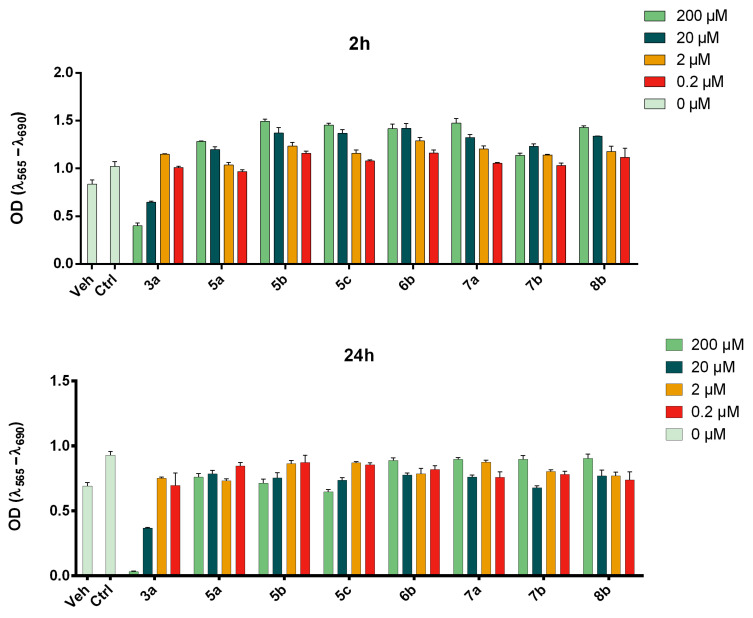
Cytotoxicity of target compounds obtained by adding MTT to fresh culture media.

Once we found that the release of H_2_S from the tested compounds could interfere with the readout of the MTT assay to measure cytotoxicity, we slightly changed the experimental protocol to minimize the impact of H_2_S release. Thus, instead of directly adding MTT to the culture media treated with the compounds, we removed the media 2 or 24 h after treatment, washed the cells with PBS, and then we added MTT dissolved in fresh culture medium. The results of these experiments are reported in Figure 4.

Following the new protocol, we still observed absorbance levels above control at 2 h, while at 24 h we observed absorbance signals below or comparable to those of control. The results confirmed the low cytotoxicity and slight cytoprotective effects of the thiourea and thioamide derivatives observed using the previous protocol. The effects of the thioamide derivatives **7a**,**b**, **8b**, and **3a** were comparable to those obtained with the previous protocol, while the effects of the thioureas, especially **5a** and **6b**, were significantly lower.

**Figure 5:**
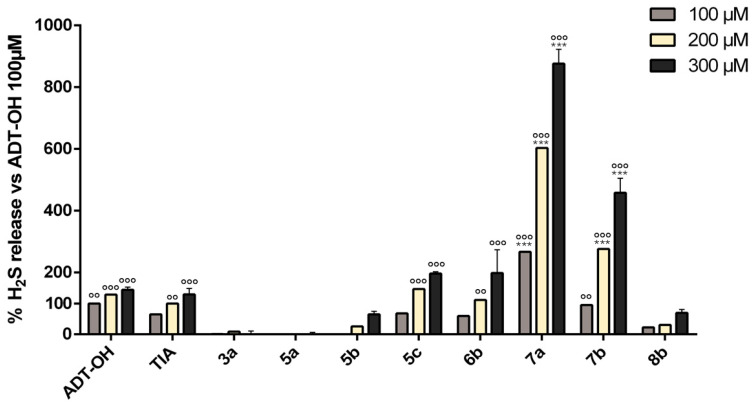
Cumulative H_2_S formation (expressed as area under the curve of WSP-1 fluorescence in the recording time) produced by the incubation of vehicle or different concentrations of the tested compounds. Data are expressed as mean ± SEM, with ADT 100μM H_2_S release set as 100%. One- way ANOVA post-test Bonferroni has been applied to calculate the significance level (*** *p* < 0.001 vs. ADT reference compound; °° *p* < 0.01, °°° *p* < 0.001 vs. vehicle).

Next, we assessed the intracellular H_2_S release of thioureas and thioamides in HepG2 cells. The compounds were tested at different concentrations (100–300 μM) to evaluate if the release was dose-dependent. We also assayed ADT-OH and TIA as reference H_2_S-donors, and **3a** as a negative control. The results were normalized to the fluorescence signal obtained with 100 μM ADT-OH. We found that adding the vehicle to HepG2 cells caused a slight increase in fluorescence signal, likely due to the endogenous production of H_2_S. The addition of ADT-OH led to a statistically significant increase in fluorescence at the tested concentrations, clearly indicating the formation of H_2_S (*p* < 0.01 vs vehicle, Figure 5). At the same time, TIA induced a statistically significant increase in fluorescence at 200 and 300 μM. As expected, **3a** induced a fluorescence signal comparable to that of the vehicle at the tested doses. Considering the thiourea derivatives, we note that **5a** induced a fluorescence signal comparable to vehicle, thus suggesting that **5a** did not release H_2_S. In contrast, **5c** and **6b** induced a statistically significant increase in the fluorescent signal at 200 and 300 µM, indicating an increase in the intracellular level of H_2_S (*p* < 0.001 vs. vehicle, Figure 5). As for the thioamide derivatives, incubation of compounds **7a** and **7b** caused a significant fluorescence signal increase at all tested concentrations. In contrast, **8b** induced a dose-dependent increase in the fluorescent signal, but it was not statistically significant. Furthermore, **7a** and **7b** at 200 and 300 μM increased the intracellular level of H_2_S more than the reference H_2_S donor ADT (*p* < 0.001 vs. ADT, Figure 5).

Among the studied compounds, thioamides **7a** and **7b** showed very high levels of H_2_S release in HepG2 cells, while thioureas **5c** and **6b** showed levels of H_2_S release similar to those of ADT-OH.

At the end of this preliminary biological characterization, compounds **7b** and **8b** emerged for their slightly different profiles: **8b** was the most potent FPR2 agonist in this study, but had a limited ability to release H_2_S. In comparison, **7b** can release a higher amount of H_2_S than **8b**, but it was less potent than **8b** as an FPR2 agonist.

Next, to assess the therapeutic potential of combining FPR2 agonism with H_2_S release, we evaluated the neuroprotective and anti-inflammatory effects of our hybrid compounds in mouse primary microglial cells. Since no examples of compounds with a similar profile are reported in the literature, compounds **7b** and **8b** were progressed to the next step of the study.

### 3.4. Effect of Compounds ***8b*** and ***7b*** on NO and Cell Death in Mouse Primary Microglial Cells

We assessed the neuroprotective potential of compounds **8b** and **7b** by evaluating their effect on NO production and cell viability in mouse primary microglial cells under basal conditions and after LPS stimulation.

NO is a multifunctional signaling molecule synthesized by nitric oxide synthases (NOS) and plays a key role in maintaining physiological homeostasis and modulating inflammatory responses. In chronic neuroinflammation, activated microglia become major sources of NO overproduction within the brain. Excessive NO levels can worsen neuroinflammation, leading to neuronal death and tissue damage [57]. NO and H_2_S may interact with each other: NO may upregulate H_2_S production and H_2_S may inhibit NO production [58]. It has been reported that H_2_S inhibits iNOS expression and NO production in RAW264.7 macrophages stimulated with LPS [59].

**Figure 6:**
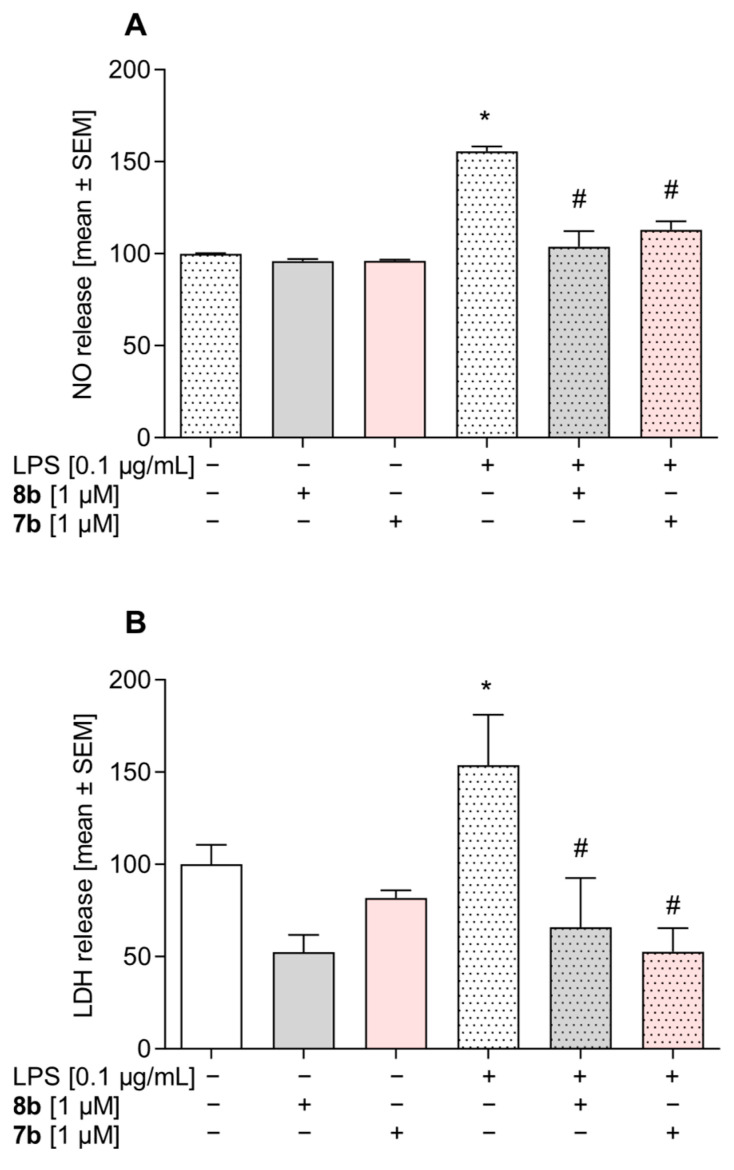
Effect of **8b** and **7b** on nitric oxide (NO) release (**A**) and cell death (lactate dehydrogenase; LDH; (**B**)) in mouse primary microglial cells. Cells were pretreated for 1 h with **8b** or **7b** (1 µM), and then stimulated for 24 h with LPS (0.1 μg/mL). The results are expressed as the mean ± SEM percentage of the control (vehicle-treated cells). *n* = 2–4 in each experiment. * *p* < 0.05 vs. control, # *p* < 0.05 vs. LPS group.

Indeed, stimulation of microglia cells with LPS (0.1 µg/mL) induced a significant increase in NO release (*p* < 0.0001, Figure 6A). We found that the pretreatment with **8b** and **7b** (1 μM) effectively attenuated the LPS-induced NO production (*p* < 0.0001, and *p* = 0.000152, respectively) (Figure 6A). Interestingly, neither compound increased NO levels under basal conditions, suggesting that compounds **7b** and **8b** have neuroprotective effects.

Next, we assessed the effects of the two compounds on cell death under basal conditions and after LPS stimulation. As LPS impairs membrane integrity, we selected the lactate dehydrogenase (LDH) assay, a well-established assay for quantifying cell death associated with plasma membrane disruption (necrosis or late-stage apoptosis). Under basal conditions, none of the tested compounds elicited a notable increase in LDH release, suggesting that the compounds did not impact cell viability. Stimulation of the microglial cultures with LPS (0.1 µg/mL) of the microglial cultures induced significant cell damage, with a pronounced increase in LDH release (*p* = 0.034, Figure 6B). Pretreatment with **8b** and **7b** (1 μM) effectively mitigated the LPS-induced cytotoxicity (*p* = 0.0025; *p* = 0.0009, respectively; Figure 6B), demonstrating their protective effects.

### 3.5. Effect of Compounds ***8b*** and ***7b*** on the Levels of Pro- and Anti-Inflammatory Factors in Mouse Primary Microglial Cells

To elucidate the anti-inflammatory and pro-resolving potential of **8b** and **7b**, we evaluated their effects on the production of pro-inflammatory cytokines IL-18 and IL-6 and on the production of the anti-inflammatory cytokine IL-10 under basal conditions and after LPS stimulation.

**Figure 7:**
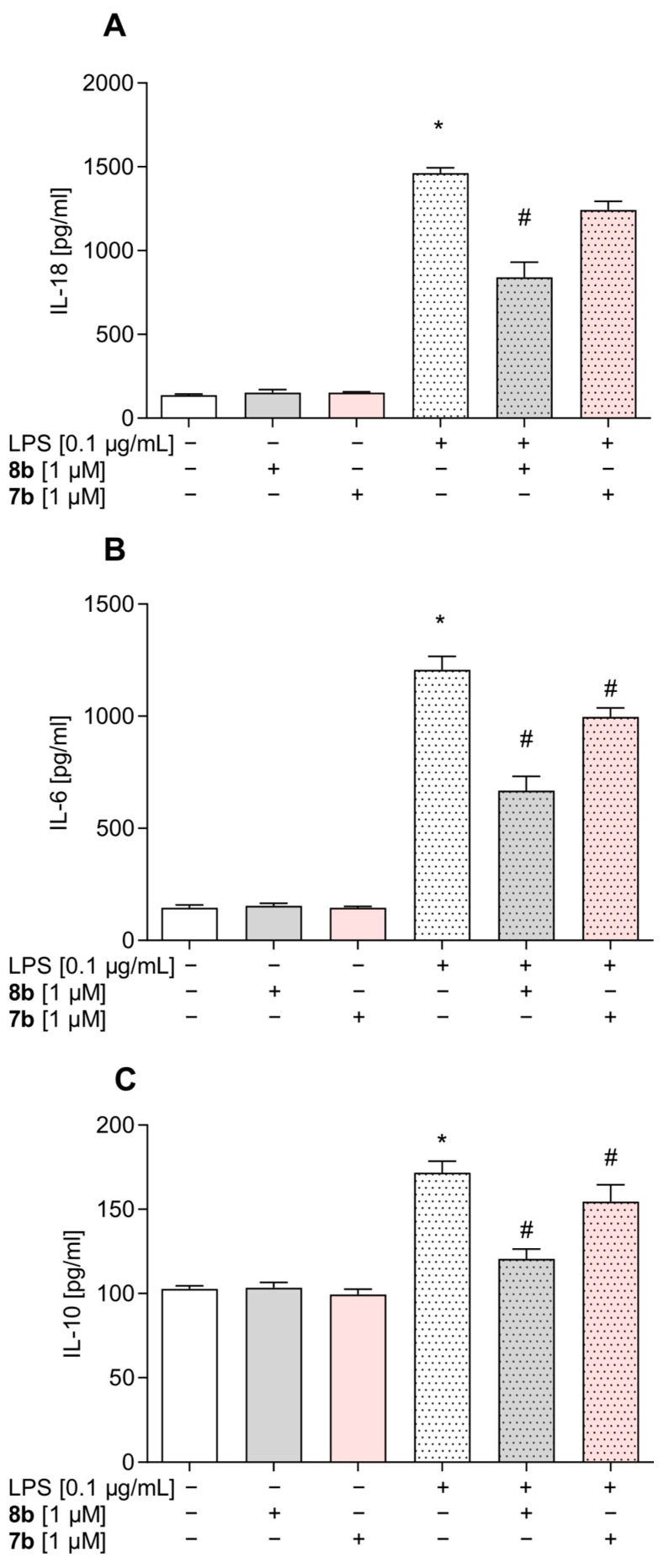
Effect of **8b** and **7b** on interleukin 18 (IL-18; pg/mL; (**A**)) interleukin 6 (IL-6; pg/mL; (**B**)), and on interleukin 10 (IL-10; pg/mL; (**C**)) levels in mice primary microglial cells. Cells were pretreated for 1 h with **8b** and **7b** (1 µM), and then stimulated for 24 h with LPS (0.1 μg/mL). The results are expressed as the mean ± SEM percentage of the control (vehicle-treated cells). *n* = 2–4 in each experiment. * *p* < 0.05 vs. control, # *p* < 0.05 vs. LPS group.

As shown in Figure 7A–C, stimulation with LPS (0.1 μg/mL) significantly increased IL-18, IL-6, and Il-10 levels in the medium of microglial cultures (*p* < 0.0001; *p* < 0.0001; *p* < 0.0001; respectively). Pretreatment with **8b** and **7b** (1 μM) was able to counterbalance the LPS-induced increase for all of the selected cytokines (**8b**: IL-18: *p* = 0.003301; IL-6: *p* = 0.000259; IL-10: *p* = 0.000087; Figure 7A–C; **7b**: IL-6: *p* = 0.029961; IL-10: *p* = 0.035994; Figure 7A–C), thus providing additional evidence that the compounds have anti-inflammatory and pro-resolving properties.

Although neither compound was able to completely counteract the effect of LPS on IL-18 and IL-6 production, compound **8b** appears to be more effective than compound **7b**. A two-step pathway may be particularly relevant for the observed effects of compound **8b**: it involves first inhibiting the phosphorylation of the p65 NF-κB subunit, followed by the inhibition of the NLRP3 inflammasome [60,61,62]. Notably, it has been reported that H_2_S may exert anti-inflammatory effects by modulating the NF-κB/NLRP3 pathways in several cell types and tissues [63,64]. Similarly, FPR2 agonists achieve their anti-inflammatory effects by modulating the same pathways [20,65,66].

## 4. Conclusions

Neurodegenerative disorders are characterized by complex etiology because different factors contribute to their progression, and the discovery of effective drugs in this area is still a significant challenge. To address such complex etiology, the scientific community is pursuing the strategy of designing multitarget-directed ligands to intervene in different nodes in the pathological network to delay or even stop the progression of the disease. Following this approach, we designed, synthesized, and biologically characterized a set of hybrid molecules that combine the ability to activate FPR2 and release H_2_S. Starting from our potent FPR2 agonists **3a**–**c**, which feature a urea and amide function that we conveniently converted into the corresponding thiourea or thioamide function that can release H_2_S.

This structural modification had a different impact on FPR2 activity: it led to a substantial decrease in agonist potency in the case of the thiourea derivatives, while it increased FPR2 potency in the case of thioamide derivatives, as compared to the ureidopropanamides **3a**–**c**. Both thiourea and thioamide derivatives release H_2_S in aqueous media and intracellularly. The amount of released H_2_S was structure-dependent, with the thioamide derivatives having a higher release in HepG2 cells than thioureas. The thioamides **8b** and **7b**, having different combinations of FPR2 agonist potency and H_2_S release, were further evaluated in mouse primary microglial cell cultures stimulated with LPS. Both compounds exhibited protective properties, counterbalancing the effects of LPS stimulation on cell viability and NO production. Interestingly, both compounds had anti-inflammatory and pro-resolving effects, re-equilibrating the levels of pro-inflammatory (IL-18 and IL-6) and anti-inflammatory (IL-10) cytokines after LPS stimulation. To our knowledge, compounds **8b** and **7b** are the first molecules with simultaneous FPR2 agonist activity and H_2_S release. Although compounds **8b** and **7b** have shown promising results in primary microglial cells, further studies are required to assess their translatability potential in vivo in animal models of neurodegenerative disorders and to elucidate the molecular mechanisms of the observed antioxidant, anti-inflammatory, and pro-resolving properties.

## Data Availability

The original contributions presented in this study are included in the article/Supplementary Materials. Further inquiries can be directed to the corresponding author(s).

## Supplementary Materials

The following supporting information can be downloaded at: https://www.mdpi.com/article/10.3390/antiox14070827/s1, Figure S1: dose–response curve of compound **7b**; Figure S2: dose–response curve of compound **8b**.
