# Supplementary figures and images for: Hydrogen Sulfide (H2S)-Donating Formyl Peptide Receptor 2 (FPR2) Agonists: Design, Synthesis, and Biological Evaluation in Primary Mouse Microglia Culture
Source: Antioxidants (Basel). 2025 Jul 4;14(7):827. doi: 10.3390/antiox14070827 (PMC12291851; doi:10.3390/antiox14070827)

Figure S1 supplement

**A**

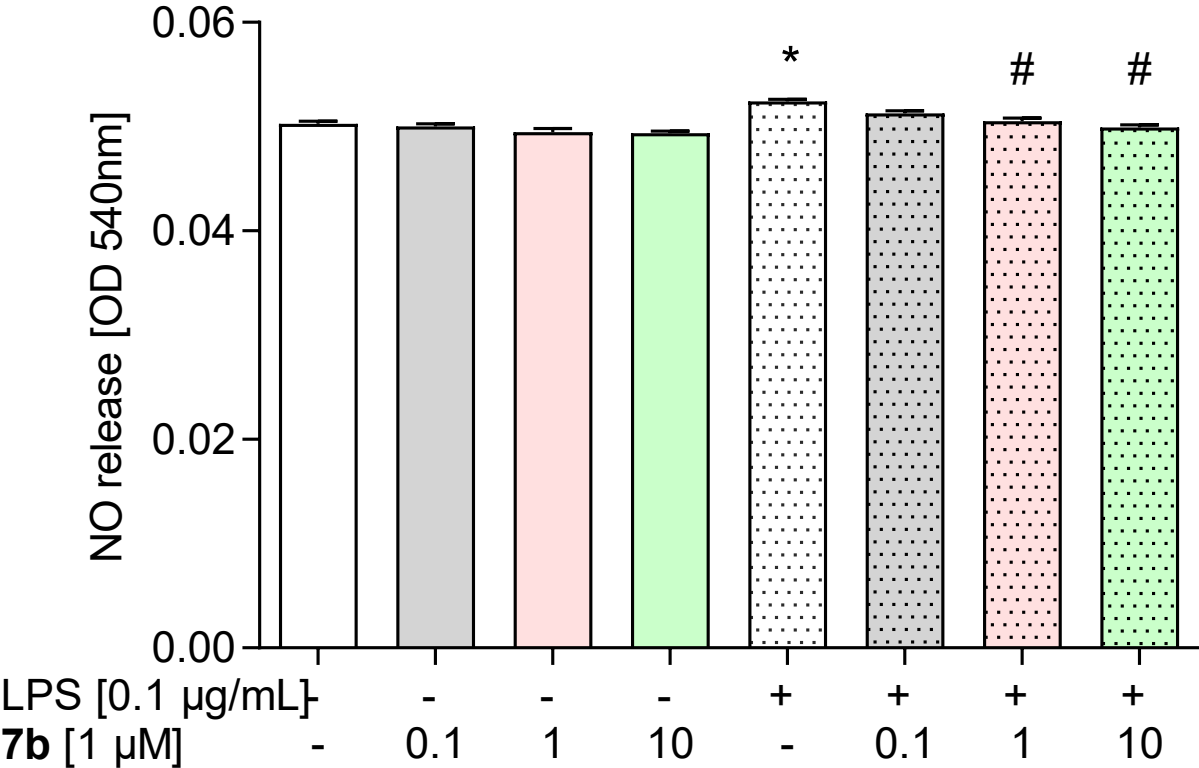

**B**

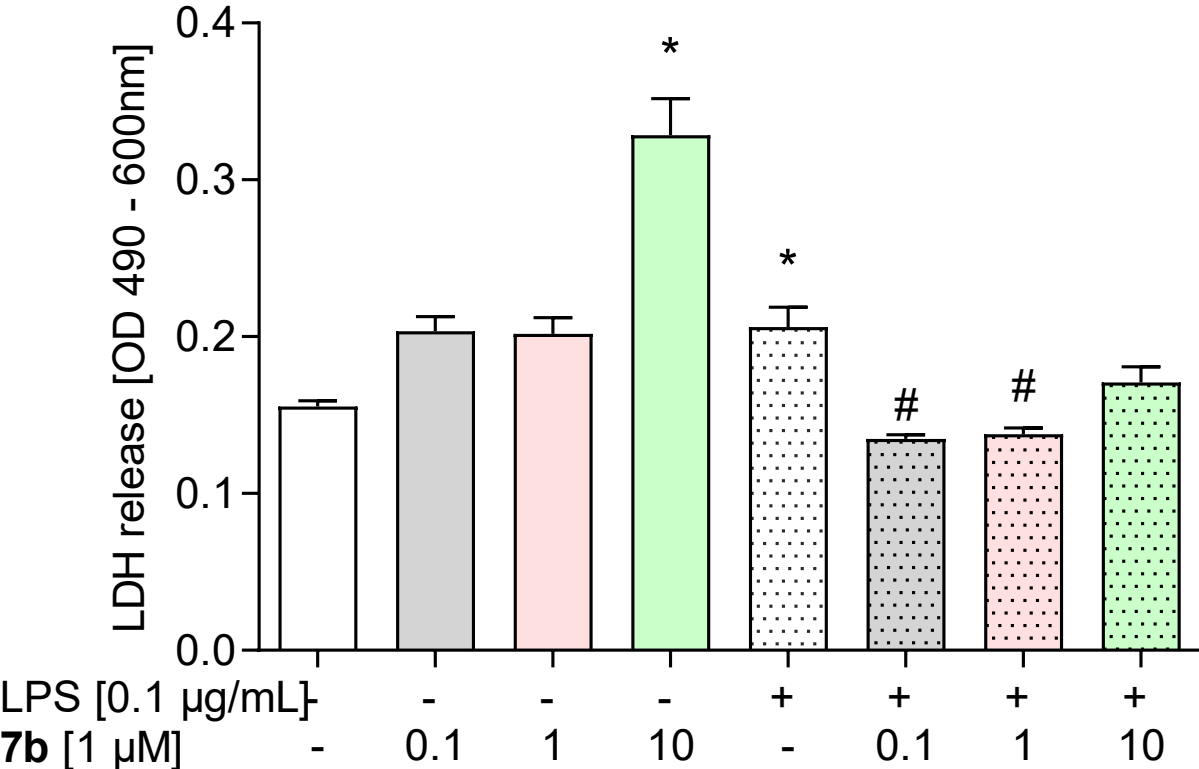

Figure S2 supplement

A

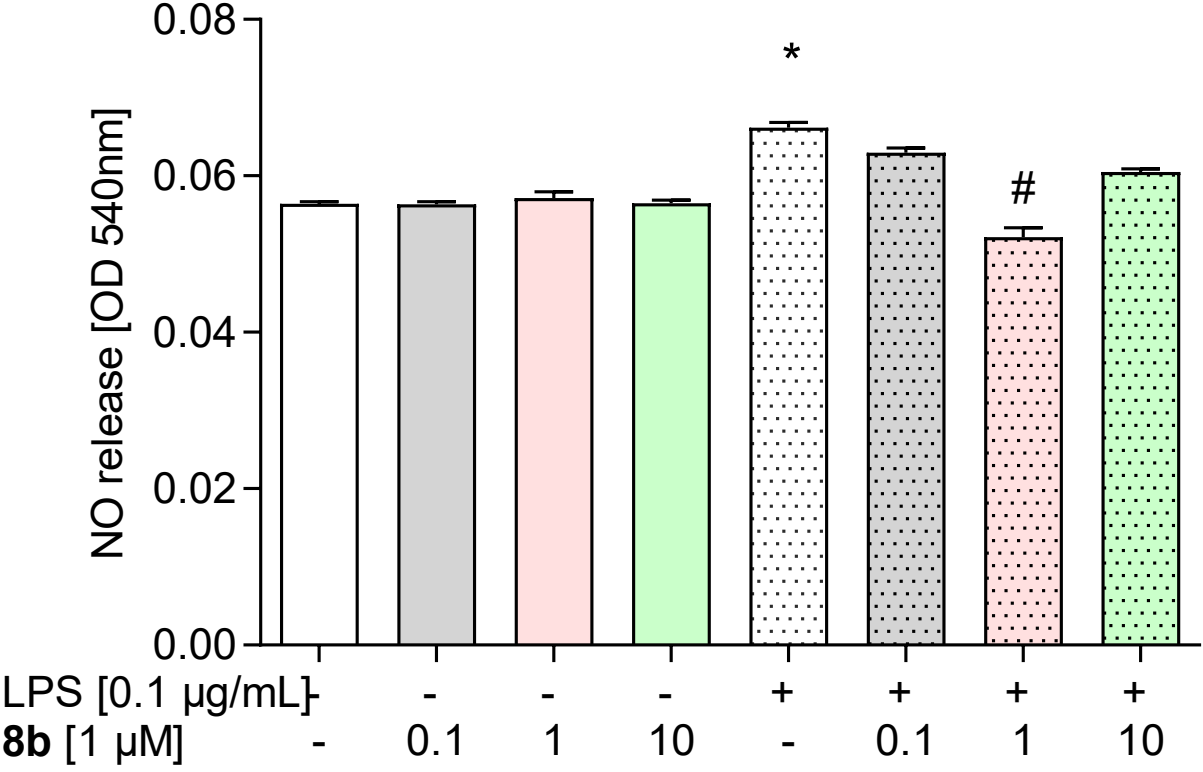

B

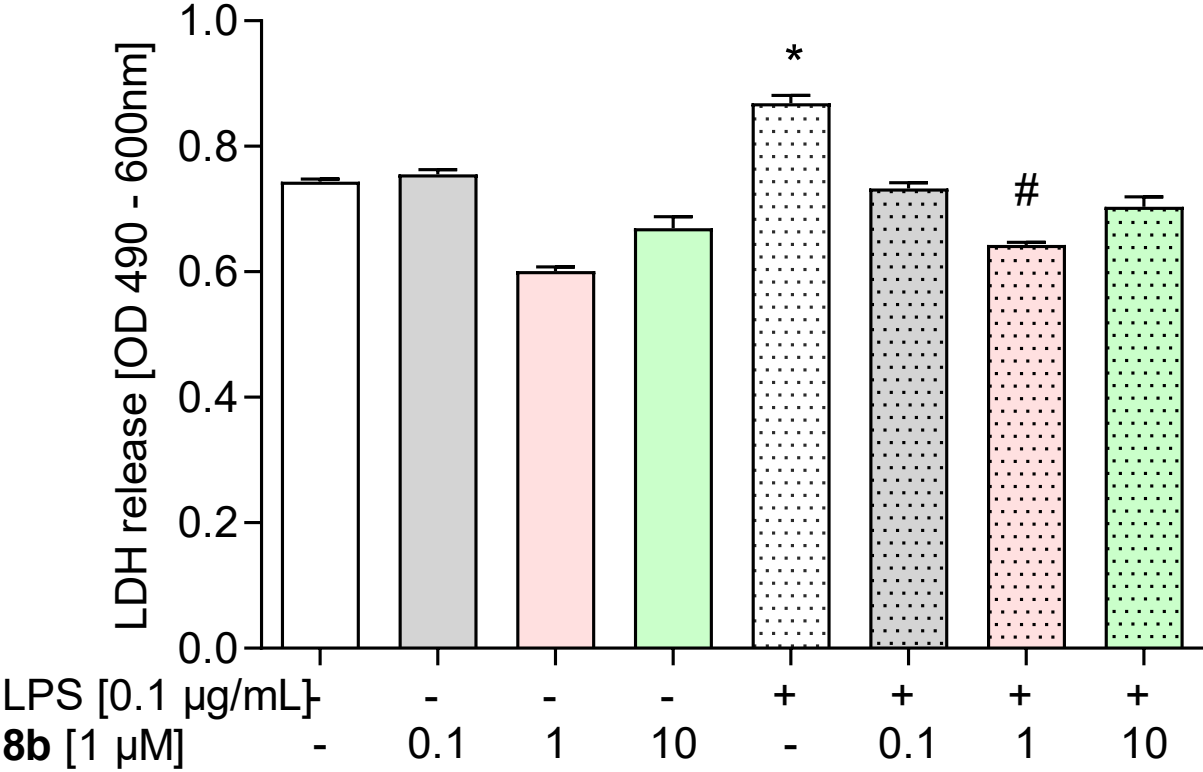

Supplement: Supplementary file 1 [file antioxidants-14-00827-s001.zip › antioxidants-3712158-supplementary.pdf]
